# The language we borrow

**DOI:** 10.1017/S1478951526103320

**Published:** 2026-07-23

**Authors:** Vangipuram Harshil Sai

**Affiliations:** Academic Section - Department of Undergraduate Education, All India Institute of Medical Sciences New Delhi, India

The first time I heard the phrase, “We’ll keep fighting,” I thought only of its kindness. My consultant had just explained that the latest scan showed progression despite chemotherapy. The patient sat in silence, his eyes fixed on the CT images as though they no longer belonged to him, while his wife continued smoothing the edge of her dupatta between her fingers, her face calm even as her hands betrayed the anxiety she refused to show. My consultant waited for a moment before leaning forward and offering the familiar reassurance. “We’ll keep fighting.”

Her hands went still. The patient nodded. Nothing about the prognosis had changed, yet the room seemed to settle around those 3 words, as though they had done what medicine could not.

As a medical student, I found that reassuring. The smallest word in the sentence – *we* – made illness feel a little less lonely. I had not yet asked what, exactly, was being shared. I admired how naturally experienced doctors found the right words when certainty began to fade, and I gradually realized that no one had ever taught them to speak this way. The phrase passed quietly from one generation to the next, absorbed through ward rounds, clinics, and difficult conversations, until it no longer sounded borrowed. It became part of what caring sounded like, long before I had any reason to wonder if it was.

Over the following months, I heard the phrase everywhere – in oncology clinics after disappointing scans, outside operating theatres when surgery carried more uncertainty than reassurance, and in intensive care units where every conversation balanced hope against honesty. “We’ll try another treatment.” “We’ll get through this.” “We’ll keep fighting.” The words shifted slightly each time they were used. The pronoun almost never did.

It took me much longer to notice something else: patients rarely answered in the same way. “I’m tired.” “I don’t think I can do another cycle.” “I just want to be at home.” At first, this seemed like nothing more than an accident of conversation, but once I noticed it, I could not stop noticing it. Doctors reached for the plural without thinking; patients almost always returned to the singular. For weeks, I wondered why.

The answer came on an afternoon that appeared entirely ordinary. A man with metastatic lung cancer had been coming to our department for nearly a year, long enough that we no longer needed to introduce ourselves. The nurses knew he liked his tea strong, with the milk poured in first. He thanked them with the same quiet formality every visit, always chose the chair by the window, and left the one by the door for his wife, who never sat until he had settled himself first. His scans had grown worse with each visit, but he still greeted everyone by name before he sat – including me, though I had done nothing to earn it.

That afternoon went much like the others. Blood tests were reviewed, symptoms discussed, and the latest scan opened on the screen, until almost without anyone noticing, the conversation reached the question no one wanted to ask aloud. What now?

My consultant leaned forward. “We’ll keep fighting.”

The patient smiled. It was not the smile of someone dismissing a cliché, nor the tired smile of someone who no longer believed. If anything, it carried an air of affection – as though he understood exactly why those words had been chosen. He lifted his teacup, took one last sip, and set it down carefully on the small wooden table between them, where months of visits had left a pale, round mark from cups set down repeatedly in almost the same place. Then he looked up.

“Doctor,” he said softly, “you all go home after this.”

No one spoke, although outside the room, the clinic carried on as usual – a porter pushed a trolley down the corridor, a nurse called the next patient’s name, a telephone rang somewhere – while inside, his sentence remained suspended in the silence.

When the clinic ended that evening, we did exactly what he had described. The nurses handed over to the night staff, my consultant dictated his letters and went home, and I walked back to my hostel, where lecture notes and exams awaited with the ordinary weight of student life. He went home too, but he carried something else entirely: nausea that would return before dinner, the tablets his wife would count out and set beside his bed, and the stubborn, quiet way his daughter would search once more for a trial none of us truly believed would change anything.

He did not challenge our compassion. He had named its limits, saying aloud what had been true in every consultation I had sat through without once hearing it.

In the weeks that followed, I paid less attention to the words themselves and more to what they were trying to do. Medicine often instinctively reaches for the plural because good care is rarely the work of one person – decisions are shared, treatment takes a team, and when recovery comes, it belongs to patients, families, nurses, therapists, pharmacists, and doctors alike. But serious illness resists being shared completely. No matter how closely we walk beside someone, some things remain entirely theirs: the pain that wakes them before dawn, the fatigue that turns a staircase into a mountain, and the uncertainty that settles over a house while everyone waits for the next scan. We step into our patients’ lives; they go on living inside their illness long after we have stepped back. That may be why patients so often speak in the singular – not refusing company, only naming where the illness actually lives.

Near the end of my posting, another family arrived after learning that further treatment was unlikely to help. The same hush filled the room, but this time, my consultant chose different words. “Let’s talk about what matters most to you now,” he said. “Whatever you decide, we’ll walk through it together.”

I noticed what had shifted. He no longer promised a shared battle or a burden split evenly. He was offering something that medicine can honestly give, even when it can no longer offer a cure: not shared suffering, but a steady presence.

I still hear the old phrases in hospital corridors, and sometimes they reach my own lips before I have chosen them. They belong to the teachers whose compassion shaped the doctor I hope to become, and I would not want to lose the generosity that first drew me to their words. What has changed is not the pronoun. It is the meaning I hear inside it now.

Even now, when I think back to that afternoon, I do not first recall the scan or the exact words that were spoken. I remember a man setting his teacup down on the pale, worn circle of a small wooden table after months of returning to the same room, and I remember that when the consultation ended, we all went home – carrying, as we always did, a little of his language and none of his illness.

